# Periodontal Disease Status among Adults from South Africa—Prevalence and Effect of Smoking

**DOI:** 10.3390/ijerph16193662

**Published:** 2019-09-29

**Authors:** Usuf Chikte, Carla Cruvinel Pontes, Innocent Karangwa, Faheema Kimmie-Dhansay, Rajiv T. Erasmus, Andre P. Kengne, Tandi E. Matsha

**Affiliations:** 1 Department of Global Health, Stellenbosch University, Cape Town 8000, South Africa; pontescarla@hotmail.com; 2 Division of Epidemiology and Biostatistics, Stellenbosch University, Cape Town 8000, South Africa; ikarangwa@uwc.ac.za (I.K.); faheemakimmie@gmail.com (F.K.-D.); 3 Division of Clinical Pathology, Stellenbosch University, Cape Town 7505, South Africa; rte@sun.ac.za; 4 Non-Communicable Diseases Research Unit, South African Medical Research Council, Cape Town, South Africa; andre.kengne@mrc.ac.za; 5 SAMRC/CPUT/Cardiometabolic Health Research Unit, Department of Biomedical Sciences, Faculty of Health and Wellness Science, Cape Peninsula University of Technology, Bellville, Cape Town 7535, South Africa; matshat@cput.ac.za

**Keywords:** periodontal disease, non-communicable diseases, oral health

## Abstract

Periodontal diseases are among the six most prevalent non-communicable diseases (NCDs) worldwide, constituting a burden for oral and general health. There is a shortage of epidemiological data on periodontal diseases in Africa. The aim of the present cross-sectional study was to present the periodontal status and cotinine levels of a South African population of adults. This study included individuals living in the Belville South area. Bleeding on probing (BOP) and pocket depth were recorded for each tooth, and clinical attachment loss (CAL) was recorded as the highest score per sextant. Cotinine levels were measured in ng/mL. A total of 951 individuals were included. More than one third of all subjects had BOP. Regarding pocket depth, over 50% of the subjects had shallow pockets (4–5 mm), and almost 6% had deep pockets. CAL ≥ 4 mm was present in 40.1% of the subjects. Males presented worse periodontal conditions than females. In total, 52.7% of the participants had serum cotinine levels of ≥15 ng/mL. Cotinine levels had no effect on periodontal variables. Periodontal diseases were highly prevalent, and periodontal conditions were worse in males. Preventive and restorative public health programs are required to improve oral health in this population.

## 1. Introduction

Periodontal diseases are among the six most prevalent non-communicable diseases (NCDs) worldwide [1]. They constitute a burden for oral health and have the potential to affect general health through the low-grade chronic inflammation and bacterial pathogens [2]. Though severe forms of periodontal disease are the major cause of tooth loss in adults worldwide, they remain neglected in public health policies in several countries. Because periodontal diseases share several risk factors with other NCDs, their prevention may have an impact that goes beyond the oral cavity [3].

Smoking is one of the strongest risk factors associated with the development and progression of periodontal disease [4,5]. According to the World Health Organization, each year, tobacco exposure leads to more than seven million deaths worldwide [6]. Cotinine is an alkaloid tobacco product and the main nicotine metabolite. Because of the longer half-life of cotinine (10–30 h) as compared to nicotine (30 min), cotinine has been used a reliable biomarker for tobacco exposure in the literature [7]. Though cotinine can be measured in samples of saliva, urine and serum, the latter presents the longest half-life [8]. Cotinine serum levels between 10 and 20 ng/mL are compatible with smoking and have been associated with an increased severity of periodontitis in a variety of studies [9,10,11,12]. The cut-off value of 15 ng/mL was used in the current study to differentiate between smokers and non-smokers based on previous studies on serum cotinine and periodontal disease [8,9]. 

The prevalence of periodontal disease differs among population groups worldwide, which is probably related to socio-behavioral and environmental factors [13]. There is a shortage of epidemiological data on the prevalence of adult periodontitis in South Africa, a country composed of population groups with diverse demographic profiles. The last national oral survey in the country took place around 30 years ago [14]. 

## 2. Purpose of the Study

The aim of the present study was to report the prevalence of periodontal disease in an adult population group from South Africa in relation to serum cotinine levels. The findings from the present epidemiological study can contribute to the creation and implementation of public health policies.

## 3. Materials and Methods

This cross-sectional epidemiological study is part of the ongoing Cape Town Vascular and Metabolic Health (VMH) study, which has been described in detail previously [15]. The study was approved by the Ethics Committee of the Faculty of Health and Wellness Sciences of the Cape Peninsula University of Technology (N14/01/003a) and conducted in accordance with the Declaration of Helsinki. Each of the participants gave written consent in order to partake the study. A consecutive sampling technique was used to recruit participants over 18 years of age living in the Belville South area in Cape Town (South Africa) from 2010 to 2014, an area characterized by low socio-economic status and mixed ancestry, as previously defined in terms of the South African Population Registration Act (Act No. 30 of 1950, repealed in 1991) [16]. The inclusion criteria included individuals with mixed ancestry older than 18 years living in the Belville South area. Individuals younger than 18 years, that required prophylactic antibiotics, with cancer, with intellectual disabilities, undergoing renal dialysis, were pregnant or who were too sick or unable to give written consent were excluded. 

### 3.1. Laboratory Investigations

A fasting blood sample was collected and kept on ice until being transported (within six hours) to an accredited laboratory for processing. Blood samples were processed for a measurement of serum cotinine levels through a chemiluminescent assay (Immulite 1000, Siemens), as described previously [16].

### 3.2. Clinical Examination

A portable dental chair and a portable overhead LED light were used by calibrated dental examiners to record the clinical periodontal variables. Standard infection control measures were used according to the Center for Disease Control [17]. The examination was conducted according to guidelines from the World Health Organization [18]. Bleeding on probing was recorded for each tooth as the presence or absence of bleeding after gentle periodontal probing around each tooth circumference. For pocket depth, each tooth was probed in its whole circumference, and the highest score was recorded. Finally, clinical attachment loss (CAL) was recorded with a periodontal probe as the highest score for each sextant.

### 3.3. Statistical Analysis

Periodontal variables were presented as percentage prevalence or average and standard deviation in relation to age group, gender and cotinine. Our study followed the guidelines from WHO; however, because of the small sample size of the oldest group (≥75 years), it was combined with the 65–74 group. Pocket depth categories included 0–3, 4–5, and ≥6 mm, but due to the small sample size of the latter, it was incorporated into the 4–5 mm group. Similarly, attachment loss categories above 4 mm were all combined for the analysis. For subject level analysis, chi-square tests were employed; for tooth and sextant level analysis, Mann–Whitney and Kruskal–Wallis tests were employed. Data were analyzed using the statistical package STATA/SE 10.0 (Stata Corp LP, College Station, TX, USA).

## 4. Results

A total of 1888 individuals were recruited, of which 859 (45.2%) were completely edentulous; another 78 patients were excluded as a result of incomplete data recording. The final number of subjects included in the periodontal clinical examination was 951; 70% (*n* = 668) were females, and 30% were males (*n* = 668). The sample sizes of the age group were 102 (<24 years, 10.7%), 238 (25–34 years, 25%), 219 (35–44 years, 23%), 244 (45–54 years, 25.7%), 101 (55–64, 10.6%) and 47 (≥65 years, 4.9%).

### 4.1. Cotinine

Cotinine levels were categorized into two groups: <15 ng/mL (compatible with non-smoking) and ≥15 ng/mL (compatible with smoking). In total, 52.7% of the participants of the study had serum cotinine levels ≥ 15 ng/mL, while 47.3% had levels below 15 ng/mL. There were no associations between cotinine categories, gender groups, and age groups in our study (Table 1, *p* > 0.05).

### 4.2. Gingival Bleeding

More than two thirds of all subjects (68.3%; *n* = 650) had bleeding on probing, and the average number of teeth with bleeding was 2.7 ± 3.4. There was a higher percentage of males with gingival bleeding as compared to females (73.9% and 66.0%, respectively; *p* = 0.02). Males also presented a higher average number of teeth with bleeding than females (3.0 ± 3.6 vs. 2.5 ± 3.3., respectively; *p* = 0.009).

**Figure 1:**
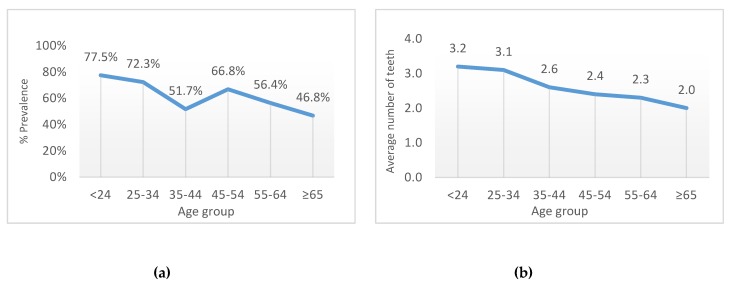
Prevalence of bleeding on probing according to age group (**a**) and the average number of teeth with bleeding on probing according to age group (**b**).

The prevalence of gingival bleeding decreased with increasing age, going from 77.5% of subjects under 24 years to 46.6% of subjects 65 years and above (*p* < 0.001; Figure 1a). The average number of teeth with gingival bleeding was 3.2 for the youngest group (<24 years), and it decreased with aging to 2.0 in the oldest group (*p* = 0.007; Figure 1b). 

In our study, cotinine categories were not associated with bleeding on probing (Table 2; *p* > 0.05).

### 4.3. Pocket Depth (PD)

In total, 56.7% (*n* = 539) of the studied sample presented periodontal pockets 4 mm and above, while 43.3% (*n* = 412) had no pockets. On average, each participant had 2.1 ± 3.1 teeth with periodontal pockets.

**Figure 2:**
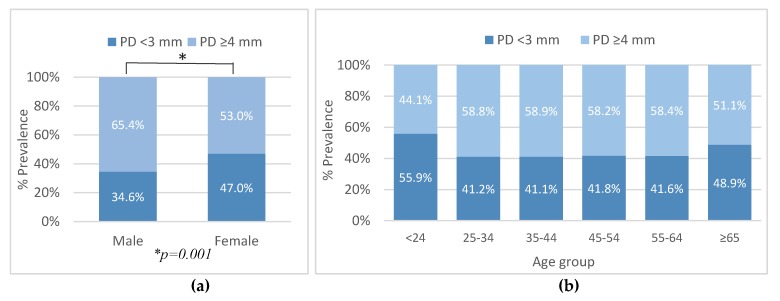
Distribution of pocket depth (PD) categories in relation to gender (**a**) and age group (**b**).

There was a higher percentage of females with no periodontal pockets in comparison to males (47.0% vs. 34.6%, respectively; *p* = 0.001; Figure 2a). The prevalence of pockets was higher in the male group as compared to females (65.4% vs. 53.0%, respectively; *p* = 0.001; Figure 2a).

When compared to males, females had a higher average number of teeth with no pockets (16.0 ± 6.8 vs. 14.7 ± 7.8, respectively; *p* = 0.03) and a lower number of teeth with periodontal pockets (1.9 ± 2.9 vs. 2.7 ± 3.4, respectively; *p* = 0.001). On the subject level, the prevalence of pockets was not associated with aging (Figure 2b; *p* > 0.05). 

**Figure 3:**
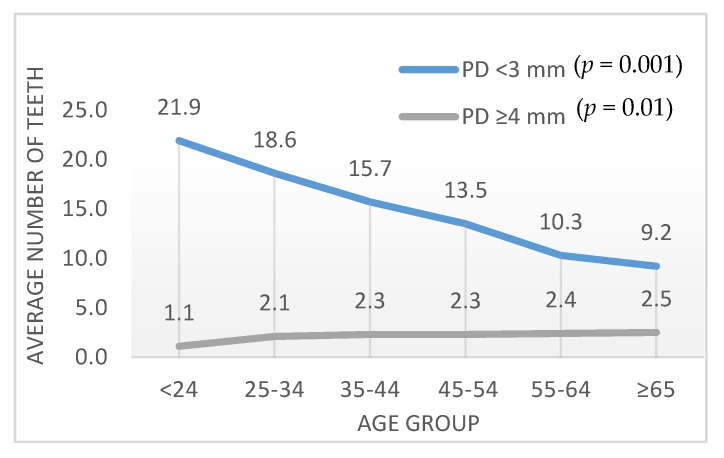
Average number of teeth with pocket depth (PD) below 3 mm and above 4 mm in relation to age group.

On the tooth level, the average number of teeth with no periodontal pockets decreased from 21.9 ± 4.5 (<24 years) to 9.2 ± 6.3 (≥65 years; *p* = 0.001; Figure 3). The average number of teeth with periodontal pockets increased from 1.1 ± 1.8 in the youngest group to 2.5 ± 3.7 in the oldest group (*p* = 0.01; Figure 3).

Cotinine categories were not associated with probing pocket depth (Table 2; *p* > 0.05).

### 3.4. Attachment Loss (AL)

In total. 40.2% of the sample had attachment loss (AL) ≥ 4 mm. The average number of sextants with AL < 3 mm was 4.3 ± 1.9 and 0.7 ± 1.1 for AL ≥ 4 mm. 

**Figure 4:**
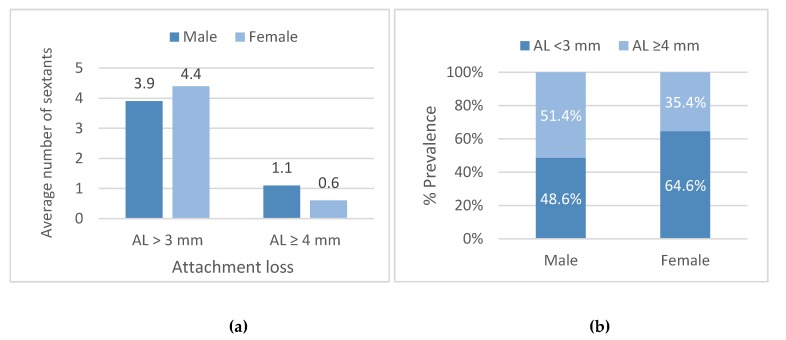
Attachment loss (AL) presented as the average number of sextants in relation to gender (**a**) and % prevalence in relation to gender (**b**).

On average, females had more sextants with AL < 3 mm than males (4.4 ± 1.8 vs. 3.9 ± 2.0, respectively; *p* = 0.001) and fewer sextants with AL ≥ 4 mm (0.6 ± 1.0 vs 1.1 ± 1.3, respectively; *p* < 0.001; Figure 4).

On the subject level, when comparing males to females, males presented a lower prevalence of AL < 3 mm (48.6% vs. 64.6%, respectively; *p* < 0.001) and a higher prevalence of AL ≥ 4 mm (51.4% vs. 35.4%; respectively; *p* = 0.001; Figure 4).

**Figure 5:**
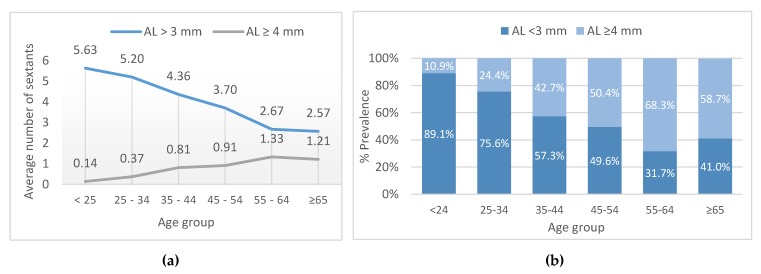
Attachment loss (AL), presented as the average number of sextants in relation to age group (**a**) and % prevalence in relation to age group (**b**).

In relation to age groups, AL < 3 mm—measured as the average number of sextants per subject—decreased with age (from 5.63 ± 0.90 for the youngest group to 2.57 ± 2.0 for the oldest; *p* = 0.001; Figure 5). On the other hand, the average number of sextants with AL ≥ 4 mm increased with age, from 0.14 ± 0.50 to 1.21 ± 1.4 (*p* < 0.001; Figure 5).

The relative frequency of subjects with AL < 3 mm decreased with age, from 89.1% in the youngest group to 41% in the oldest group (*p* < 0.001. Figure 5). The prevalence of AL ≥ 4 mm increased with age, affecting 10.9% of the youngest group and 58.7% of the oldest group (*p* < 0.001).

There was no association between cotinine categories and a loss of attachment in the studied sample (Table 2; *p* > 0.05).

## 5. Discussion

The present epidemiological study provides data on the periodontal status and exposure to tobacco for a South African adult population with a mixed ancestry background, a group hitherto underexplored. The results showed that about two thirds of the participants had gingival bleeding, more than half had periodontal pockets, and nearly 40% presented a loss of attachment. Females had better periodontal conditions than males, and periodontal health deteriorated with aging. In this sample, serum cotinine levels compatible with smoking were present in nearly 53% of the sample. However, cotinine levels were not associated with the periodontal variables.

The last South African national survey (1988/1989) reported that only three percent of subjects with a mixed ancestry background presented gingival bleeding, extremely different from the prevalence of almost 60% in the current study [14]. This discrepancy may be partly attributed to differences in the methodology. While the CPITN—based on six index teeth and a hierarchical score structure that combines gingival bleeding, calculus, and periodontal pockets [18]—was used for the national survey, in the current study, all teeth present were examined, and gingival bleeding and periodontal pockets were recorded as independent variables. Previous studies have pointed out the complexity of comparing periodontal variables according to different recording methods as well as the limitations of the CPITN index [19]. 

While the prevalence of gingival bleeding in our study was three times higher than the 22.5% prevalence reported for the U.S. adult population, it is in accordance with other upper-middle income countries such as Brazil (68.9%) [20]. Gingival bleeding decreased with aging in the current study, which could have been due to the high level of tooth extraction being the most common dental treatment for this low-income population, as previously highlighted by Van Wyk and Van Wyk (2004) and confirmed by our results (45.2% of edentulousness) [21].

The high prevalence of periodontitis in our study (nearly 60%) is in accordance with findings from other African countries like Kenya and Tanzania, both with a prevalence above 50% [22]. A comparison of our findings to the national survey from 1989 for adults 35–44 years-old shows that the prevalence of periodontitis has almost doubled for this age group, from 29.7% to 51.6% [14].

The traditional association between pocket probing depth and age reported in the literature was only statistically significant on the tooth level in the current study [23,24]. It can be assumed that the early extraction of teeth with periodontitis and/or caries changes the scenario of cumulative destruction, leading to a less clear linear correlation with age. The true prevalence of periodontitis might be underestimated due to high extraction rates [21]. The prevalence of attachment loss ≥4 mm in the current study (40.2%) is in accordance with previous results from other African countries, such as Uganda (44.3%) and Sudan (51.2%), and increased with aging [22].

The worse periodontal conditions reported for males in the present study are in agreement with previous studies from the United States [25], Philippines [26], Brazil [20], and from a global meta-analysis [27]. Several factors can contribute to the observed gender related differences, including the regulation of genes related to immune and inflammatory responses and a poorer attitude towards health associated with the male gender [28].

Cotinine has been used as a biomarker for smoking with serum levels between 10–20 ng/mL being compatible with smoking and associated with increased severity of periodontitis [9,10,11,13]. To our knowledge, there are few studies globally and no published studies in Africa that have evaluated cotinine levels in relation to periodontal status. In the current study, the difference using 10 ng/mL, 15 ng/mL, and 20 ng/mL as cotinine thresholds was minimal. Hence, we decided to use 15 ng/mL as the cut-off value, a value which has been widely used in the literature [7,8,9]. 

Despite the fact that smoking has been a well-recognized risk-factor for periodontitis, cotinine was not associated with periodontal status in this study [11]. It is possible that, because the study was based on households and due to the fact that nearly half of the participants had cotinine levels compatible with active smoking, the other half of the participants could have been exposed to passive smoking. Hence, the true biological effects of tobacco exposure on periodontal disease could have been masked due to passive smoking. In this study, we only evaluated serum nicotine out of at least 93 potentially hazardous chemicals present in cigarette smoke identified by the US Food and Drug Administration [29]. In order to aid the understanding of the effects of tobacco exposure on oral health, future studies should also focus on other harmful chemicals present in tobacco smoke.

The high prevalence of periodontal disease and tobacco exposure in the studied population, combined with the high level of edentulousness, points to the lack of effective intervention and prevention programs to decrease the burden of periodontal diseases and promote tobacco cessation. As a non-communicable disease (NCD) that shares risk factors and social determinants with other NCDs associated with high mortality, such as cardiovascular diseases, diabetes and cancer, periodontal diseases constitute not only a burden for the health of each individual but also a burden for public health, with high morbidity, costs and socio-economic impact [30]. Action is needed to provide access to professional intervention and the creation of public policies to promote periodontal health, in accordance to WHO and UN strategies for NCDs through the common risk factor approach [31].

### Limitations of the Study

Because the amount of female participants was high in this study (70%), the possibility of sampling bias during recruitment of subjects cannot be excluded. The clinical examinations were conducted during working hours. This possibly hindered employed males from participating. It would have been interesting to look at oral hygiene and bacterial plaque in relation to periodontal variables. However, due to time and financial constraints, those variables were not recorded.

## 6. Conclusions

The studied population has a high prevalence of gingival bleeding, periodontal pockets and attachment, as well as a high prevalence of cotinine levels compatible with smoking. Males presented worse periodontal status than females. In general, the periodontal conditions of this specific sample of a mixed ethnic background deteriorated since the last national survey in 1988/89 and requires urgent attention from a public health and policy perspective. Findings from this report suggest that periodontal disease and smoking are a burden for this South African population. Action is needed through public health programs for tobacco cessation and prevention, as well as the treatment of periodontal diseases in order to improve oral and general health and wellbeing, according to the common risk factor approach for NCDs.

## Figures and Tables

**Table 1:** Serum cotinine categories according to gender and age group, presented as % (count).

	< 15 ng/mL	< 15 ng/mL
Gender		
Male	45.6% (129)	54.4% (154)
Female	48.1% (321)	51.9% (347)
Age group		
< 24	42.2% (43)	57.9% (59)
25–34	48.7% (116)	51.3% (122)
35–44	45.7% (100)	54.3% (119)
45–54	50.8% (124)	49.2% (120)
55–64	47.5% (48)	52.5% (53)
65–74	40% (19)	60% (28)
		
Total	47.3% (450)	52.7% (501)

**Table 2:** Cotinine categories in relation to gingival bleeding, pocket depth, and attachment level.

	< 15 ng/mL	< 15 ng/mL
Gingival bleeding		
Subjects with BOP	46.5% (302)	53.6% (348)
Subjects without BOP	49.2% (148)	50.8% (153)
Pocket depth		
< 3 mm	46.8% (193)	53.2% (257)
≥ 4 mm	47.7% (219)	52.3% (282)
Attachment level		
< 3 mm	47.1%(267)	47.8% (300)
≥ 4 mm	52.9% (182)	52.2% (199)
